# NOTCH3 Gene Polymorphism is Associated With the Prognosis of Gliomas in Chinese Patients

**DOI:** 10.1097/MD.0000000000000482

**Published:** 2015-03-06

**Authors:** Zhipeng Shen, Xuwei Hou, Bo Chen, Peng Chen, Qing Zhang

**Affiliations:** From the Department of Neurosurgery, Children's Hospital, Zhejiang University School of Medicine, 57 Zhugan Lane, Hangzhou 310003, P.R. China (ZS); Hangzhou people hospital, Huansha Road 261, Hangzhou 310006, P.R.China (XH); Department of Rehabilitation, Hangzhou Hospital of Zhejiang Corps, Chinese People's Armed Police Forces, 86 Jiangnan Avenue, Hangzhou 310051, P.R. China (BC); Department of Neurosurgery, The Second Affiliated Hospital, Zhejiang University School of Medicine, 88 Jiefang Road, Hangzhou 310009, P.R. China (PC); Department of Cardiology, Children's Hospital; Zhejiang University School of Medicine, 57 Zhugan Lane, Hangzhou 310003, P.R. China (QZ).

## Abstract

Recent studies show that NOTCH3 is involved in the glioma development and it is also a prognostic factor for glioma patients. However, the gene polymorphism of NOTCH3 in gliomas prognosis remains unknown.

A total of 266 patients were enrolled into this study. The NOTCH3 gene polymorphism at 3 loci, including C>T polymorphism at nucleotide 381, C>A polymorphism at 474 and G>A polymorphism at 684 were determined. All patients received the surgical treatment and/or chemotherapy and/or radiotherapy.

We found that the 684G>A polymorphism affects the tumor NOTCH3 expression level and is closely associated with a higher tumor grade, poorer tumor differentiation, and karnofsky performance score in these glioma patients. More importantly, the 684G>A polymorphism is significantly associated with the prognosis of these patients regardless of their treatment manner.

Our study indicates that the NOTCH3 gene 684G>A polymorphism may be used as a prognosis marker for gliomas.

## INTRODUCTION

Glioma is the most common central nervous system tumor comprising approximately 80% of malignant brain tumors. In spite of the improvements in diagnosis and treatment, the prognosis of glioma remains very poor (with a 5-year survival rate less than 30%).^1^ Recent studies revealed that the genetic polymorphisms of certain genes are associated with the susceptibility to gliomas and also affect its prognosis.^2–4^

NOTCH3 is a member of the NOTCH signaling pathway which includes NOTCH ligands, NOTCH receptors, and transcription factors. ^5^ NOTCH signaling plays a critical role in the proliferation and survival of stem/progenitor cells in a number of tissues, including the central and peripheral nervous systems. NOTCH signaling activation has been demonstrated to be involved in carcinogenesis.^6–8^ A recent study showed that NOTCH3 activation promotes invasive glioma formation in gliomas in a tissue site-specific manner.^9^ NOTCH3 is not expressed in normal brain tissues, but is highly expressed in astrocytomas.^10^ NOTCH3 is a prognostic factor that promotes glioma cell proliferation, migration and invasion.^11^

The genetic polymorphism of NOTCH3 regulates its protein expression. Several genetic polymorphisms of NOTHC3 gene have been reported, including C>T polymorphism at nucleotide 381, C>A polymorphism at 474, and G>A polymorphism at 684. Although the role of NOTCH3 in gliomas has been reported, however, little is known about the relation between *NOTCH3* gene polymorphisms and the clinical feature as well as the prognosis of glioma patients. In this study we found that the NOTHC3 gene polymorphism can affect the clinical feature and prognosis of glioma patients. Our data suggest that the NOTHC3 gene polymorphism may serve as a molecular marker for gliomas.

## METHODS

### Patients

This study included 266 glioma patients diagnosed and treated at our hospital. The tumor specimens were obtained during operation and were classified according to the current WHO system.^12^ The tumor stage and were acquired from medical record of each patient. All participants were genetically unrelated ethnic Han Chinese people. The information regarding the demographic factors, family history of cancer, smoking status, karnofsky performance score (KPS scores) and other health characteristics were obtained. These patients underwent the surgical resection and adjuvant therapy (radiotherapy, chemotherapy or both) from March 2005 to August 2011. Tumor tissue samples are obtained during the surgery for histological analyses. All the patients were followed up every 3 months. During the follow-up period, overall survival was measured from diagnosis to death or the last follow-up. Patients, who died of diseases not directly related to their gliomas or due to unexpected events, were excluded from this study. This study was approved by the institutional review boards of our hospital. All patients gave written informed consent to participate in the present study.

### NOTCH3 Genotyping

DNA was extracted from peripheral whole blood using a Qiagen DNA Isolation Kit (Qiagen, Valencia, CA). The NOTCH3 sequencing was performed as previously described.^13^ The subsequent step was the labeling reaction carried out using ABI BigDye™ Terminator (BDT) v3.1 Cycle Sequencing chemistry. Cycling protocol for BDT reaction: (96 °C, 1 min)—1 cycle, (96 °C, 10 s; 50 °C, 5 s; 60 °C, 4 min)—30 cycles, and (4 °C, 5 min; 10 °C, 5 min; and 4 °C, 2 min)—1 cycle. Following this, the samples were purified and concentrated using ethanol precipitation with sodium acetate, EDTA and ethanol.

### Western Blot Assay

The tumor samples were collected from each patient during surgical treatment. Samples were homogenized and lysed. Extracts were resolved on SDS-polyacrylamide gels followed by transfer to nitrocellulose membranes. Proteins were resolved by electrophoresis on 8–12% sodium dodecyl sulfate–polyacrylamide gels and transferred by electroblotting to polyvinylidene difluoride membranes. The membranes were blocked with 5% nonfat dry milk and incubated overnight at 4 °C with the anti-NOTCH3 (Santa Cruz, 1:1000), anti-Osteopontin (OPN, Santa Cruz, 1:1000), anti-NF-kB (Santa Cruz, 1:1000) and anti-GAPDH (Santa Cruz, 1:2000) antibodies. The anti-human IgG was used as negative control. Immunolabeling was detected using the enhanced chemiluminescent Reagent (Amersham Biosciences, Buckinghamshire, UK).

### Statistical Analysis

The *χ*^2^ analysis or Fisher's was used to analyze the clinical characteristics of glioma patients based on the NOTCH3 genotypes. To determine the odd ratios of each genotype, multivariate logistic regression analysis was performed, with the adjustment of their clinical characteristics. A forward stepwise (Likelihood Ratio) procedure was used for multivariable analysis. The overall survival (OS) rate was defined as the percentage of patients after their diagnosis or the start of treatment. The OS rate was compared by using log-rank test. We performed univariate and multivariate Cox proportional hazard regression analysis to estimate the effect of OPN polymorphisms on survival in the presence of other known prognostic factors. We calculated hazard ratios (HR) and their corresponding 95% confidence intervals (95%CI). Analyses were performed using the software SPSS 16.0 (SPSS Inc., Chicago, IL). All *P* values were two-sided, and a *P* value <0.05 was considered significant.

## RESULTS

### Clinical Features of Glioma Patients Stratified by NOTCH3 Genotypes

We analyzed the clinical characteristics of glioma patients based on their NOTCH3 genotypes. The distribution of 381C>T and 474C>A polymorphisms did not have a significantly difference when stratified by clinical characteristics (Tables 1a and 1b). However, we found that the NOTCH3 684G>A polymorphism was significantly associated with the clinical features of patients (Table 1c). The 684GG genotype is more prevalent in patients with higher tumor grade (WHO III + IV), larger tumor size (>1.5 cm), poorer differentiation, and lower KPS scores (<70). Using 684AA as the reference, multivariable regression analyses showed 684GG is correlated to a higher risk for III + IV tumor grade (odds ratio = 5.19, *P* < 0.001), larger tumor size (odds ratio = 3.37, *P* = 0.002), poorer differentiation (odds ratio = 3.40, *P* < 0.001) and lower KPS scores (odds ratio = 3.03, *P* < 0.001) after adjustment with age, sex, smoke status and family history, and histology type.

**Table 1a:**
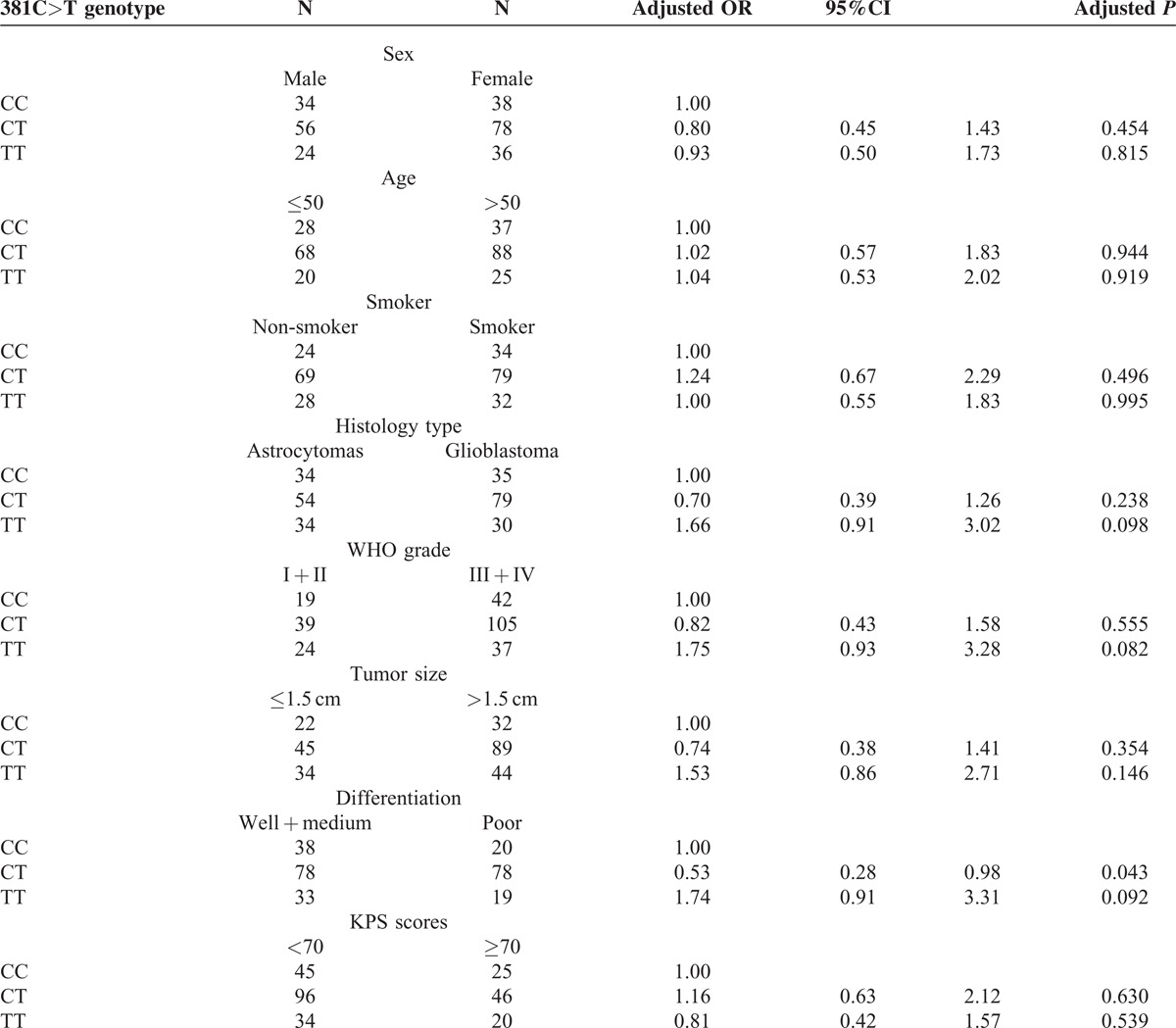
The clinical characteristics of glioma patients based on their NOTCH3 381C > T genotypes

**Table 1b:**
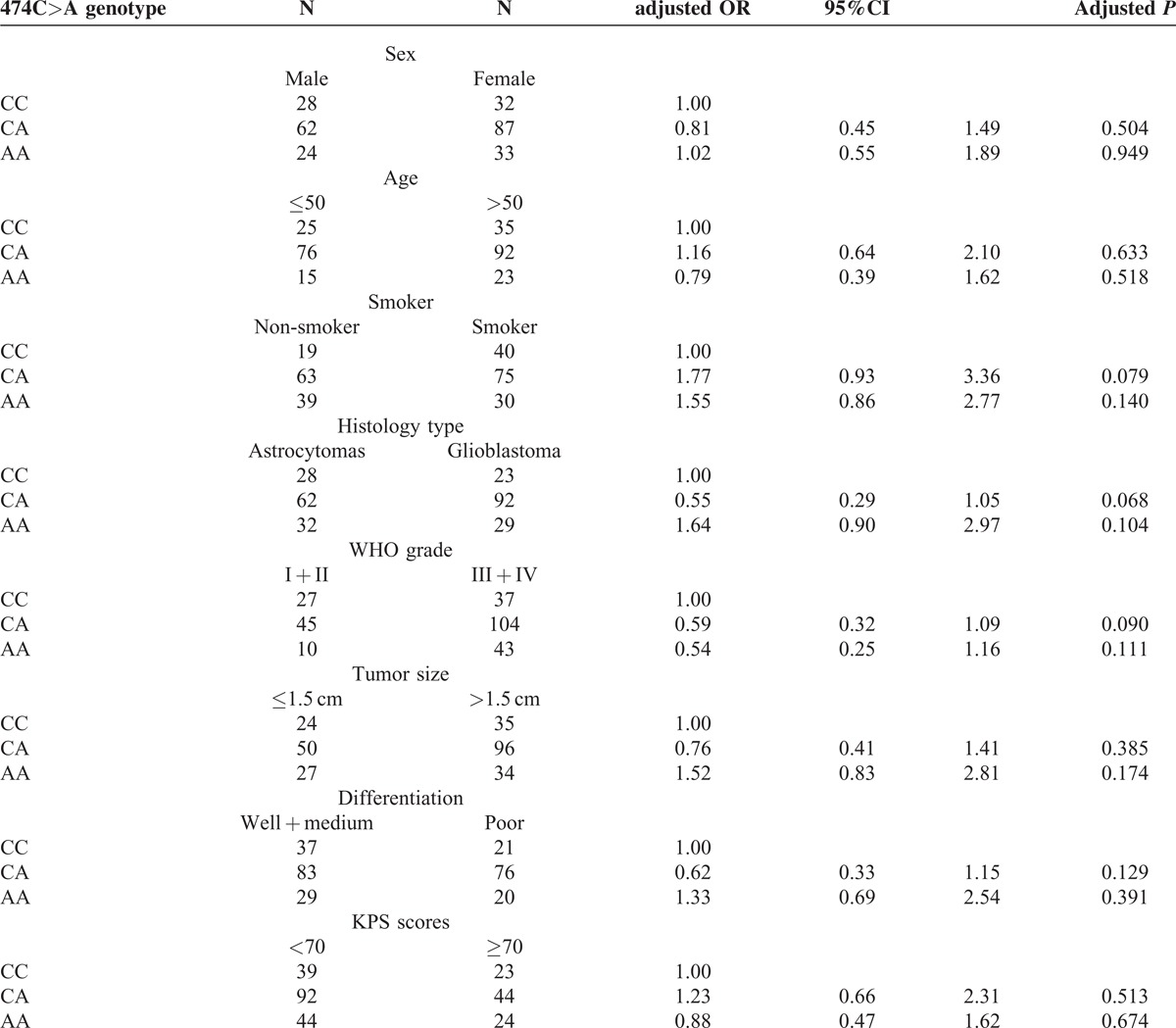
The clinical characteristics of glioma patients based on their NOTCH3 474C>A genotypes

**Table 1c:**
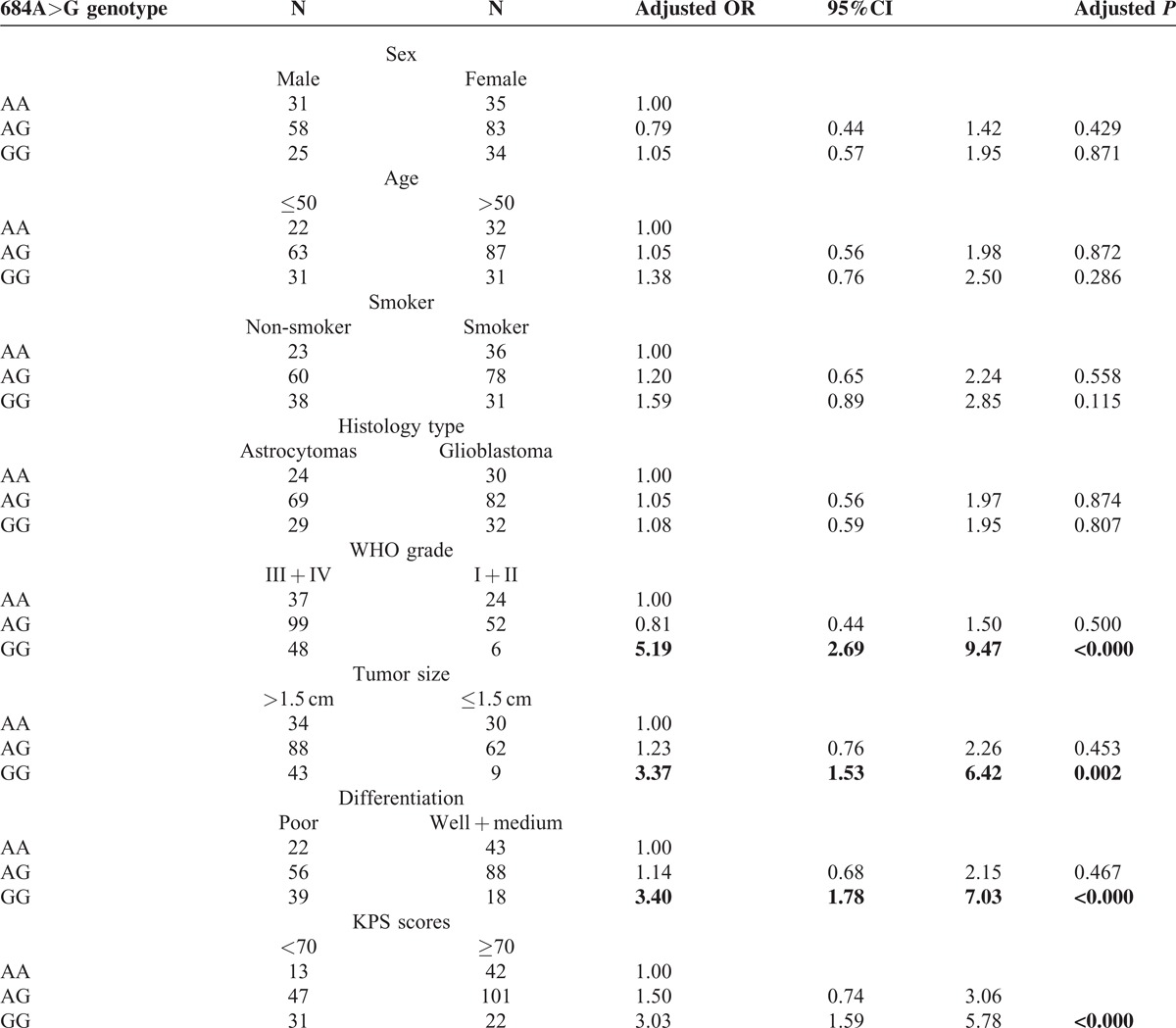
The clinical characteristics of glioma patients based on their NOTCH3 684A > G genotypes

### Western Blot for NOTCH3 From Tumor Samples

**FIGURE 1:**
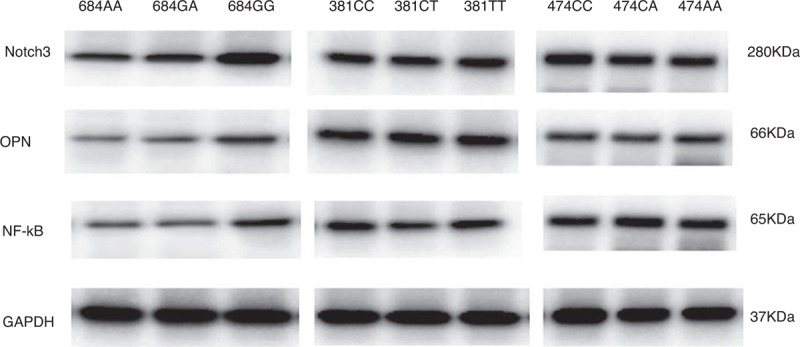
The expressions of NOTCH3 in NOTCH3 genotypes by western blot assay. We found that the 684G>A polymorphism significantly affected the tumor NOTCH3 expression levels. NOTCH3 expression is significantly higher in the tumor samples from the 684GG carriers than those from the 684GA and 684AA carriers. Meanwhile the 684G>A polymorphism also dramatically affects tumor osteopontin (OPN) and NF-kb activity. The NOTCH3 expression levels were similar among the tumor samples from the 381C>T and 474C>A genotype carriers.

The expressions of NOTCH3 in different NOTCH3 genotype carriers were detected by western blot assay. Figure 1 shows only the 684G>A polymorphism significantly affected the tumor NOTCH3 expression levels. The 684GG carriers had a higher NOTCH3 expression level than 684GA and 684AA carriers. In contrast, the 381C>T and 474C>A did not affect the NOTCH3 expression levels. Meanwhile the 684G>A polymorphism also dramatically affect tumor OPN and NF-kb expressions.

### Follow-Up Analyses

**FIGURE 2:**
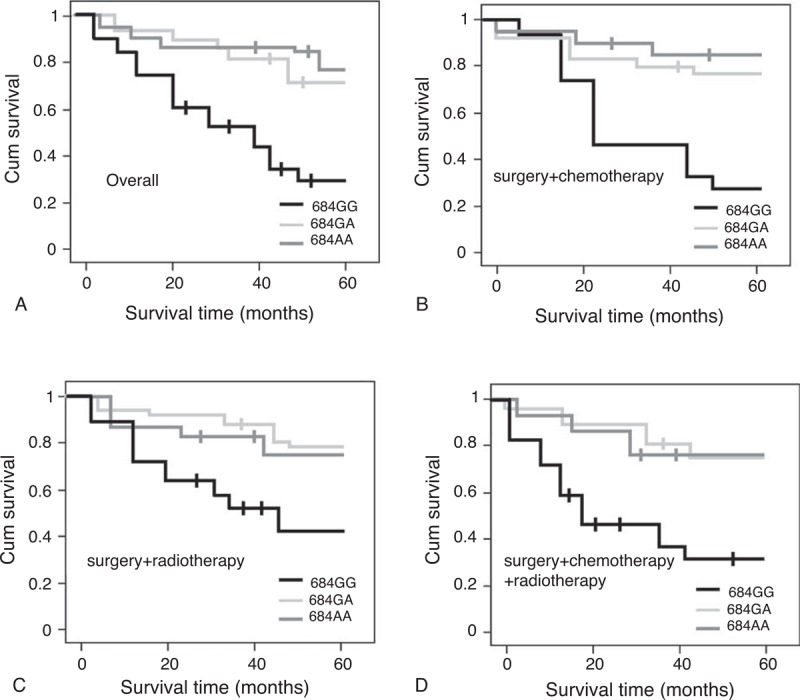
The Kaplan–Meier survival curves for glioma patients based on the 684G>A polymorphism in patients receiving different treatment. 684G>A polymorphism was the only one which can affects the overall survival rate of glioma patients. (A) shows the 684GG polymorphism was associated with significantly poorer survival of the patients (*P* < 0.001) in overall patients. (B) The effect of the 684G>A polymorphism on the survival rate in glioma patients treated with surgery + chemothreapy. (C) The effect of the 684G>A polymorphism on the survival rate in glioma patients treated with surgery + radiothreapy. (D) The survival rate of glioma patients treated with surgery + chemotherapy + radiotherapy. The 684GG was always associated with a significantly lower survival rate in these groups (all *P* < 0.001).

Of the 266 patients, 208 patients had the complete the follow-up. The log-rank test was performed to assess the effect of NOTCH3 polymorphism on the patient survival. We found that NOTCH3 684G>A polymorphism is the only one that affected the prognosis of gliomas. Overall, the 684GG polymorphism carriers had significantly lower survival rate (41.2%) than those with 684GA polymorphism (64.8%) and 684AA polymorphism (65.1%). Figure 2A shows the Kaplan–Meier survival analysis of all glioma patients, stratified by the 684G>A polymorphism. We next subgrouped all patients into 3 groups according to the treatment methods: surgery + chemotherapy, surgery + radiotherapy, and surgery + combination of chemotherapy and radiotherapy. The 684GG polymorphism always had lower survival rates compared to 684GA polymorphism and 684AA polymorphism carriers (all P < 0.001, Figure 2B, C, and D).

Univariate and multivariate Cox proportional hazards regression models were performed to estimate the crude HRs, adjusted HRs for OS rate in cases and their 95%CIs, with adjustment for age, sex, smoke status, tumor histology, size, WHO grade, and therapy status. The 684G>A polymorphism is a predictor for unfavorable prognosis of gliomas patients (HR = 3.23, 95%CI: 2.49–4.65, *P* = 0.0012).

## DISCUSSION

In the present study, we studied the association between the NOTCH3 gene polymorphism and the clinical feature and prognosis of gliomas in Chinese patients. We found that the 684G>A polymorphism affects the tumor NOTCH3 expression level and is closely associated with a higher tumor grade, poorer tumor differentiation, and KPS scores in these gliomas patients. More importantly, the 684G>A polymorphism is significantly associated with the prognosis of these patients regardless of their treatment manner. Our study indicates that the 684G>A polymorphism may be used as a molecular marker for gliomas.

The role of NOTCH signaling pathway in cancer has been reported in human colon adenocarcinoma^14^ and T-ALL cell lines.^15^ Recent studies indicate that the NOTCH signaling pathway regulates neural stem cells.^16^ The role of NOTCH pathway in central neurological system tumor is reported as well. NOTCH signaling components have been found to be deregulated in meningiomas.^17^ NOTCH activity has been observed to be critical in medulloblastoma cell line, TE671.^18^ In glioma cells, the NOTCH signaling pathway plays an important role in proliferation, stem cell maintenance, cell differentiation, and tumorigenesis.^19^

There are several members in the NOTCH family (NOTHC1-4, of which NOTCH3 in more important in glioma formation. A recent study indicates that the introduction of activated NOTCH3 into developing ocular tissues induced a number of aggressive neoplasms, including invasive gliomas arising from the optic nerve and retina, while NOTCH1 and NOTCH2 activation does not efficiently generate ocular tumors.^9^ That was the reason we selected NOTCH3 gene polymorphism in this study.

Mutations in the NOTCH3 gene cause cerebral autosomal dominant arteriopathy with subcortical infarcts and leukoencephalopathy (CADASIL) amd migraine.^20,21^ As a synonymous polymorphism, 684 G>A is thought to be non functional and does not alter coding sequences. However, recent studies reveal that the seemingly non-functional polymorphisms, like 684 G>A, could affect transcription, splicing, mRNA transport or translation, any of which could influence the resultant phenotype.^21^ In line with this finding, we found in our study that the 684G>A polymorphism can affect the NOTCH3 expression level in tumor tissues from glioma patients. The G684A alleles were observed to be significantly associated with migraine, while the 381C>T does not affect the migraine risk.^21^ A recent study reported the interaction between one of the known NOTCH3 gene polymorphism (381C>T) with 2 other genes (MTHFR C677T and ALOX5AP T2354A) to be a significant contributor to thrombotic stroke.^22^

To date, only very few studies reported the association of NOTCH3 gene polymorphism with tumor in human. A recent study showed that NOTCH3 gene is significantly overexpressed in ovarian cancer samples. Furthermore, NOTCH3 DNA copy number is positively correlated with NOTCH3 protein expression based on parallel immunohistochemistry and FISH studies in 111 high-grade tumors.^23^ However, another study NOTCH1 gene polymorphism is associated with the T type leukemia, but the genotypes of NOTCH2, NOTCH3 and NOTH4 genes are not associated with human cancers.^24^ Our study showed that the 684G>A polymorphism affects the tumor NOTCH3 expression level and is closely associated with a higher tumor grade, poorer tumor differentiation and KPS scores in these gliomas patients. More importantly, the 684G>A polymorphism is significantly associated with the prognosis of these patients regardless of their treatment manner. Our study indicates that the 684G>A polymorphism may be used as a molecular marker for gliomas.

Several limitations should be addressed in this study. The sample in this study is relatively small and limited to Chinese patients, thus the conclusion of this study warrants further confirmation in multi-ethnic population with larger scale. Secondly, we did not have in vitor study to show the effect of 684G>A polymorphism of NOTCH3 on the biological behavior of glioma tumor cells. Thirdly, the molecular mechanism under which the 684G>A polymorphism affect the gloimas risk and the prognosis in gliomas patients is not elucidated in this study.

## References

[1] Haapasalo J Hyartt A Salmi M [Diagnosis and prognosis of gliomas—current prospects of molecular diagnostics]. *Duodecim* 2014; 130:893–901. 24881141

[2] Li M Zhou Q Tu C Jiang Y A meta-analysis of an association between the XRCC1 polymorphisms and gliomas risk. *J Neurooncol* 2013; 111:221–228. 23238971 10.1007/s11060-012-1022-1

[3] Liang HJ Yan YL Liu ZM Association of XRCC3 Thr241Met polymorphisms and gliomas risk: evidence from a meta-analysis. *Asian Pac J Cancer Prev* 2013; 14:4243–4247. 23991984 10.7314/apjcp.2013.14.7.4243

[4] Assem M Sibenaller Z Agarwal S Enhancing diagnosis, prognosis, and therapeutic outcome prediction of gliomas using genomics. *OMICS* 2012; 16:113–122. 22401657 10.1089/omi.2011.0031 PMC3300066

[5] Ehebauer M Hayward P Martinez-Arias A Notch signaling pathway. *Sci STKE* 2006; 2006:cm7. 17148788 10.1126/stke.3642006cm7

[6] Rose SL Notch signaling pathway in ovarian cancer. *Int J Gynecol Cancer* 2009; 19:564–566. 19509550 10.1111/IGC.0b013e3181a12ed2

[7] Brzozowa M Mielanczyk L Michalski M Role of Notch signaling pathway in gastric cancer pathogenesis. *Contemp Oncol (Pozn)* 2013; 17:1–5. 23788953 10.5114/wo.2013.33765 PMC3685346

[8] Zhang XM Wang JX Lei XG [Regulation and mechanism of Notch signaling pathway in small cell lung cancer]. *Zhonghua Bing Li Xue Za Zhi* 2010; 39:95–99. 20388374

[9] Pierfelice TJ Schreck KC Dang L Notch3 activation promotes invasive glioma formation in a tissue site-specific manner. *Cancer Res* 2011; 71:1115–1125. 21245095 10.1158/0008-5472.CAN-10-0690 PMC3076023

[10] Xu P Yu S Jiang R Differential expression of Notch family members in astrocytomas and medulloblastomas. *Pathol Oncol Res* 2009; 15:703–710. 19424825 10.1007/s12253-009-9173-x

[11] Alqudah MA Agarwal S Al-Keilani MS NOTCH3 is a prognostic factor that promotes glioma cell proliferation, migration and invasion via activation of CCND1 and EGFR. *PLoS ONE* 2013; 8:e77299. 24143218 10.1371/journal.pone.0077299 PMC3797092

[12] Chen HJ Zheng ZC Yuan BQ The effect of galectin-3 genetic variants on the susceptibility and prognosis of gliomas in a Chinese population. *Neurosci Lett* 2012; 518:1–4. 22465244 10.1016/j.neulet.2012.02.065

[13] Roy B Maksemous N Smith RA Two novel mutations and a previously unreported intronic polymorphism in the NOTCH3 gene. *Mutat Res* 2012; 732:3–8. 22373597 10.1016/j.mrfmmm.2012.02.004

[14] Reedijk M Odorcic S Zhang H Activation of Notch signaling in human colon adenocarcinoma. *Int J Oncol* 2008; 33:1223–1229. 19020755 10.3892/ijo_00000112 PMC2739737

[15] Zhou L Wang DS Li QJ Downregulation of the Notch signaling pathway inhibits hepatocellular carcinoma cell invasion by inactivation of matrix metalloproteinase-2 and -9 and vascular endothelial growth factor. *Oncol Rep* 2012; 28:874–882. 22736202 10.3892/or.2012.1880

[16] Kageyama R Ohtsuka T Hatakeyama J Ohsawa R Roles of bHLH genes in neural stem cell differentiation. *Exp Cell Res* 2005; 306:343–348. 15925590 10.1016/j.yexcr.2005.03.015

[17] Cuevas IC Slocum AL Jun P Meningioma transcript profiles reveal deregulated Notch signaling pathway. *Cancer Res* 2005; 65:5070–5075. 15958550 10.1158/0008-5472.CAN-05-0240

[18] Yokota N Mainprize TG Taylor MD Identification of differentially expressed and developmentally regulated genes in medulloblastoma using suppression subtraction hybridization. *Oncogene* 2004; 23:3444–3453. 15064731 10.1038/sj.onc.1207475

[19] Kanamori M Kawaguchi T Nigro JM Contribution of Notch signaling activation to human glioblastoma multiforme. *J Neurosurg* 2007; 106:417–427. 17367064 10.3171/jns.2007.106.3.417

[20] Schwaag S Evers S Schirmacher A Genetic variants of the NOTCH3 gene in migraine—a mutation analysis and association study. *Cephalalgia* 2006; 26:158–161. 16426270 10.1111/j.1468-2982.2005.01007.x

[21] Menon S Cox HC Kuwahata M Association of a Notch 3 gene polymorphism with migraine susceptibility. *Cephalalgia* 2011; 31:264–270. 20813781 10.1177/0333102410381143

[22] Liu J Sun K Bai Y Association of three-gene interaction among MTHFR ALOX5AP and NOTCH3 with thrombotic stroke: a multicenter case-control study. *Hum Genet* 2009; 125:649–656. 19373490 10.1007/s00439-009-0659-0

[23] Park JT Li M Nakayama K Notch3 gene amplification in ovarian cancer. *Cancer Res* 2006; 66:6312–6318. 16778208 10.1158/0008-5472.CAN-05-3610

[24] Lee SH Jeong EG Yoo NJ Mutational analysis of NOTCH1, 2, 3 and 4 genes in common solid cancers and acute leukemias. *APMIS* 2007; 115:1357–1363. 18184405 10.1111/j.1600-0463.2007.00751.x

